# Plant Proteins Are Smaller Because They Are Encoded by Fewer Exons than Animal Proteins

**DOI:** 10.1016/j.gpb.2016.06.003

**Published:** 2016-12-18

**Authors:** Obed Ramírez-Sánchez, Paulino Pérez-Rodríguez, Luis Delaye, Axel Tiessen

**Affiliations:** 1 Genetic Engineering Department, CINVESTAV Unidad Irapuato, Irapuato, CP 36821, Mexico; 2 Colegio de Postgraduados, Campus Montecillo, Texcoco, CP 56230, Mexico

**Keywords:** Digital proteome, Eukarya, Evolution, Viridiplantae, Polypeptide length

## Abstract

Protein size is an important biochemical feature since longer proteins can harbor more domains and therefore can display more biological functionalities than shorter proteins. We found remarkable differences in protein length, exon structure, and domain count among different phylogenetic lineages. While eukaryotic proteins have an average size of 472 amino acid residues (aa), average protein sizes in plant genomes are smaller than those of animals and fungi. Proteins unique to plants are ∼81 aa shorter than plant proteins conserved among other eukaryotic lineages. The smaller average size of plant proteins could neither be explained by endosymbiosis nor subcellular compartmentation nor exon size, but rather due to exon number. Metazoan proteins are encoded on average by ∼10 exons of small size [∼176 nucleotides (nt)]. Streptophyta have on average only ∼5.7 exons of medium size (∼230 nt). Multicellular species code for large proteins by increasing the exon number, while most unicellular organisms employ rather larger exons (>400 nt). Among subcellular compartments, membrane proteins are the largest (∼520 aa), whereas the smallest proteins correspond to the gene ontology group of ribosome (∼240 aa). Plant genes are encoded by half the number of exons and also contain fewer domains than animal proteins on average. Interestingly, endosymbiotic proteins that migrated to the plant nucleus became larger than their cyanobacterial orthologs. We thus conclude that plants have proteins larger than bacteria but smaller than animals or fungi. Compared to the average of eukaryotic species, plants have ∼34% more but ∼20% smaller proteins. This suggests that photosynthetic organisms are unique and deserve therefore special attention with regard to the evolutionary forces acting on their genomes and proteomes.

## Introduction

The biological function and the physical structure of proteins are mainly influenced by their primary structure, *i.e.*, the total number, composition, and order of amino acid residues (aa). The chemical environment also affects the structure of a folded polypeptide, but the primary sequence is crucial for obtaining a fully functional protein. Short proteins (<200 aa) usually have limited functionalities while long proteins (>500 aa) have more options for accommodating multiple secondary structures and therefore more functional and regulatory domains [1], [2], [3]. A positive exponential relationship between protein length (PL) and number of domains (ND) has been reported for animal proteins [4].

There are significant differences in protein length among the different domains of life. Eukaryotic proteins are on average longer than bacterial proteins, and these in turn are longer than archaeal proteins [5], [6], [7]. Furthermore, eukaryotic genomes contain ∼7-fold more proteins that are on average ∼48% larger than bacterial ones [7]. There are also differences in average protein sizes among eukaryotic taxa. A negative correlation was found between protein number and protein size, revealing domain fusion and protein splitting events occurring in different eukaryotic proteomes [7]. In contrast, protein number and protein size are positively correlated within bacterial genomes [7]. The causes of protein length variability among eukaryotic phylogenetic groups are yet unknown. However, several evolutionary processes have shaped protein length: (1) endosymbiosis and migration of bacterial genes into the nucleus [8]; (2) genome duplication leading to polyploidy [9], [10]; (3) genomic reduction and selective gene loss [11]; (4) fusion of single-function proteins into multi-domain proteins [5]; (5) horizontal gene transfer [12], [13]; (6) intron gain and/or exon loss [7], [14], [15]; and (7) evolution of multi-domain proteins [4], [16], [17].

Each process has a distinct effect on the proteome (the sum of all encoded polypeptides in a genome). For example, chromosomal or genome duplications increase the total number of proteins without altering average protein size. However, long proteins (more complex genes) are more likely to be retained after whole genome duplication, probably because they are more prone to subfunctionalization and neofunctionalization [18]. On the one hand, transposon insertions and gene splitting increase the number of proteins but reduce average protein size. On the other hand, gene fusion reduces the number of proteins but increases average protein size (merged multi-domain proteins). There is evidence supporting that those balancing processes indeed occur in both directions as demonstrated by the significant negative correlation between protein size and the total number of proteins in eukaryotic genomes [7].

Plants are special eukaryotes since they are autotrophic. In comparison to fungal and animal cells, plants possess an additional cell organelle, the chloroplast, which hosts many of the unique features of photosynthetic organisms. In this article we address the following questions. (1) Are proteins from plants larger or smaller on average when compared to cyanobacterial, animal or fungal proteins? (2) How is protein size in eukaryotes correlated to exon size or exon number? (3) What is the impact on protein length of bacterial gene migration from the chloroplast to the nucleus? (4) Do proteins from different subcellular compartments have different average protein sizes? In order to answer these and other similar questions, we analyzed the proteomes of eukaryotic species that were publicly available.

## Results and discussion

### Eukaryotic proteins show a large diversity of sizes

We determined mean and median protein length in three independent proteome datasets. Datasets 1 and 2 were manually curated as reported previously [7]. Dataset 1 contains mainly fully-sequenced genomes (51 eukaryotes together with some selected prokaryotes for comparison including 24 eubacteria and 9 archaea). Their entries were filtered for non-redundancy by eliminating duplicated sequences, subsequences, alternative splicing variants, and transposons [7]. Dataset 2 was constructed from genomes available in Kyoto Encyclopedia of Genes and Genomes (KEGG) database and contains 97 archaea, 1205 bacteria, and 140 eukaryotes [7]. Dataset 3 covering a wider taxonomic range of eukaryotic species was constructed from the RefSeq release 70 (see Methods) without being filtered for redundancy. It contains 492 eukaryotes from most branches of the eukaryotic tree of life (Table 1).

**Figure 1:**
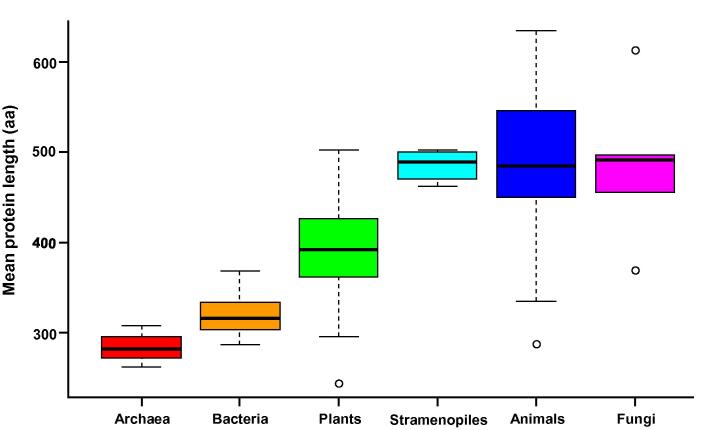
**Variation of average protein size in dataset 1** Boxplots show the distribution of protein length (aa) in different lineages (distribution of species means among the taxonomic group). Species were grouped as described previously [5]. Protein size average was calculated for the genome of each species first and then the distribution of species means among the taxonomic group was plotted. aa, amino acid.

Boxplot analysis of the curated dataset 1 revealed that archaeal and bacterial species display a narrow range of average sizes, whereas eukaryotic species have a wider range of variation (Figure 1). Genomes from prokaryotes had smaller proteins (<350 aa) than genomes from eukaryotes (>400 aa) in both datasets 1 and 2. Green plants have an average protein size that is between that of bacteria and that of non-photosynthetic eukaryotic species (Figure 1). These results were consistent across datasets 1, 2 and 3, thus demonstrating that the statistical analyses on incomplete genomes (datasets 3) are robust and minimally affected by diverse mathematical artifacts resulting from alternative splicing and limited sampling size (see Methods). Moreover, this allowed us to generalize the overall conclusion: there are remarkable differences in average protein length between prokaryotes and eukaryotes (Figure 1).

### Protein length in plants is intermediate between bacteria and animals

**Figure 2:**
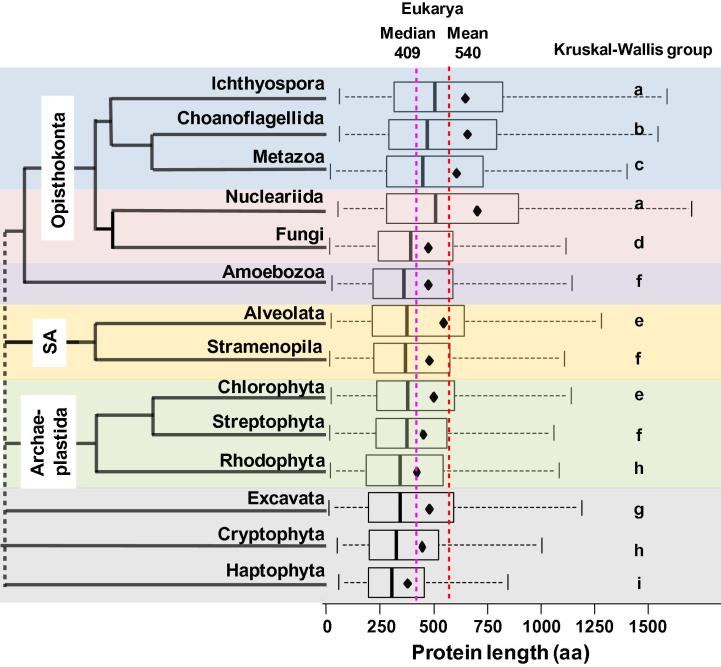
**Protein length across the eukaryotic tree of life in dataset 3** Boxplots of protein length (aa) among 14 phylogenetic groups are arranged according to evolutionary origin. Different phylogenetic clades are indicated with different color backgrounds. Solid vertical lines indicated medians, whereas diamond points indicate means of protein length. The dotted vertical lines show the mean (red) and the median (purple) length of all eukaryotic proteins, respectively. Different letters indicate significant differences between different phylogenetic groups based on Kruskal–Wallis test (*P* < 0.05).

Inspection of the proteomes from datasets 1 and 2 indicated that there were large differences in protein length across the domains of life (Figure 1) [7]. Statistical analysis of the proteomes from dataset 3 indicated that there were also significant differences in protein length between several eukaryotic groups (Table 2). Average and median protein sizes were relatively conserved among closely-related evolutionary lineages, so that differences across taxonomic groups resulted to be highly significant (*P* < 0.05; Table 2). Therefore, we grouped the organisms into the main taxonomic clades according to modern versions of the eukaryotic tree of life [19], [20], [21]. Figure 2 shows comparisons of protein length between 14 phylogenetic groups. Proteins in the Opisthokonta clade had the largest length among the eukaryotes. Among them, Ichthyosporea, Nucleariida, and Choanoflagellida had longer proteins than Metazoa, which in turn had longer proteins than Fungi (Figure 2). Protein length in the Archaeplastida clade (Rodophyta, Chlorophyta, and Streptophyta) was smaller than that in the Opisthokonta clade (Figure 2 and Table 2). Finally, Cryptophyta and Haptophyta groups had the smallest mean and median protein length among eukaryotes (Figure 2 and Table 2). Clearly, there are significant differences (*P* < 1E−16) in protein size between plants, fungi, animals, and other eukaryotes. Taken together, results from all our 3 datasets indicated that plants have an intermediate size (smaller than metazoan and fungal proteins but larger than bacterial proteins). As expected from a lognormal distribution which has a long right tail [7], differences were more remarkable for the means than for the medians.

### Plants genomes code for more proteins but their proteins are of smaller size

The number of proteins in plant species (36,795 on average in each genome) is greater than that in animal (25,189) and fungal species (9113) (Table 1). This indicates that compared to animals (Metazoa), plants (Streptophyta) had on average 46% more proteins (Table 1) but these proteins were of smaller size (Table 2). The average of metazoan proteins (595 aa) was 36% larger than the average of plant proteins (436 aa) (Table 2). The 90% percentile of the size of plant proteins was in the range of 649–877 aa, whereas in animals it was in the range of 909–1125 aa. Logarithmic normalization also confirmed that proteomes of plants have a smaller group of long proteins (>500 aa) than those of animals (Figure S1). Compared to heterotrophic fungi, photosynthetic organisms (Archaeplastida) also have smaller protein sizes (Figure 2).

We found it remarkable that plant proteins were significantly smaller than animal and fungal proteins (Figure 1, Figure 2; Table 2), which prompted us to explore the possible causes. A more detailed comparison among photosynthetic organisms revealed differences also among plant subgroups (*P* < 0.01). Green algae (Chlorophyta) had 66% fewer proteins but their proteins were 12% larger on average than other plant groups (Streptophyta and Rhodophyta) (Table 1, Table 2). The red algae *Cyanidioschyzon merolae* also had fewer but on average larger proteins (5002 proteins of size ∼504 aa) than vascular plants (>20,000 proteins of size ∼436 aa). The monocot species such as *Oryza sativa* (379–448 aa), *Zea mays* (345–402 aa), *Sorghum bicolor* (361–418 aa), and *Brachipodium distachyon* (428–457 aa), had slightly larger proteins than the dicot species, including *Carica papaya* (∼296 aa), *Medicago truncatula* (245–295 aa), and *Populus trichocarpa* (375–390 aa) in terms of the range of mean values in the 3 different datasets. Interestingly, despite having a compact genome, average protein size in *Arabidopsis thaliana* (403–410 aa) was not particularly small compared to other plants. This indicates that intergenic DNA can be expanded or contracted by several evolutionary forces without affecting average protein sizes. Arabidopsis is the best annotated plant genome and the calculated average protein size of 410 aa is larger compared to other plant genomes that have been less well annotated (*e.g.*, barrel clover or papaya with ∼296 aa). This observation supports the hypothesis that the distribution of protein sizes changes from an initially monotonic decrease function (due to random open reading frames) to a gamma function (sharp starts in the range of 1–100 aa) and then finally to a lognormal distribution (soft starts from 1 to 100 aa) as the genomes evolve or become better annotated [7].

In order to check whether proteins unique to plants are shorter than plant proteins conserved among other eukaryotic lineages, we consulted the Plant Specific Database (PLASdb) [22]. We extracted the gene IDs of the 3848 *Arabidopsis* genes that are unique to plants and analyzed the protein length distribution of that subset. On the one hand, size of the plant-specific proteins (median of 282 aa and mean of 321 aa) was significantly smaller (Wilcox test, *P* < 0.001) than that of the whole *Arabidopsis* proteome (median of 346 aa and mean of 402 aa) and the Streptophyta pan-proteome (median of 363 aa and mean of 436 aa) (Table 2). On the other hand, the size of *Arabidopsis* proteins shared among multicellular eukaryotes, *e.g.*, plants and Metazoa (median of 392 aa and mean of 458 aa) was significantly larger (Wilcox test, *P* < 0.001) than that of *Arabidopsis* and Streptophyta (Table 2). There is a significant (*P* < 0.01) length difference of 64–81 aa between plant-specific or cyanobacterial proteins and plant proteins that have orthologs in animals. These data suggest that protein size varies according to the phylogenetic lineage, evolutionary history (Figure 1, Figure 2), biological function, and cellular organization.

In order to identify the factors that determine the smaller average size of plant proteins compared to animals and fungi, we tested four possible explanations, including transposons, endosymbiosis, subcellular compartmentation, and exon structure.

### Mathematical artifacts due to transposon cannot explain the differences

The first and simplest explanation is a numerical artifact due to the high abundance of small transposons in plant genomes [23], [24]. The size distribution of proteins can be accurately approximated by several probabilistic models, with gamma (with unrestricted shape parameter) and the lognormal models [7] as the best fit. Instead of only comparing a single value (*e.g.*, mean or median), we mathematically modeled the distribution curves and calculated all percentiles in order to confirm the differences across species. Such approach allows to group samples according to the statistical test of Kruskal–Wallis (Table 2). Manual data curation and exhaustive statistical analysis, such as removal of protein redundancy (*e.g.*, dataset 1) and elimination by keywords in annotations (*e.g.*, dataset 2), led us to exclude that the results were due to the frequent appearance of only one type of protein (transposon or retroelements). We found instead a systematic shift in protein sizes across the whole range (Figure S1).

We also found that alternative splicing only marginally affected the average or median length of the proteins (Table 2 and data not shown). Similar results were obtained for given taxonomic groups no matter protein redundancy was filtered out or not (*e.g.*, datasets 1 *vs.* 3). The explanation may be simple: splicing may be universal in all eukaryotes and occurs regardless of final protein size [25], [26], [27], [28], [29]. There is no bias for alternative splicing to occur only in very small proteins or only in very large proteins [30], [31].

### Did endosymbiosis reduce the average size of plant proteins?

The second possible explanation as to why proteins are smaller in plants could be the acquisition of thousands of genes from chloroplasts after endosymbiosis. Two facts could support this hypothesis: the first one is that cyanobacterial proteins are smaller than those of eukaryotes [7], and the second one is that cyanobacteria are the ancestors of plastids in the Viridiplantae and Streptophyta groups [32]. Therefore, the intermediate size of plant proteins might arise from a massive migration of small proteins from bacterial origin (chloroplast) to the eukaryotic nucleus [33], reducing the overall average size by a dilution effect.

Based on previous data [7], an intuitive explanation can be as follows: after endosymbiosis, migration of an estimate of 3500 cyanobacterial proteins of ∼319 aa in length to a hypothetical ancient eukaryote having ∼22,900 proteins of ∼472 aa in length would lead to a new eukaryote having average protein length of ∼451 aa. However, that size is still in the range of protein size in animals and fungi. Therefore such postulation cannot explain the results observed for plants.

To follow a more robust approach, we compared protein size between *Arabidopsis* nuclear proteins and their cyanobacterial orthologs. According to previous studies [8], [34], [35], genes transferred from chloroplasts to the nucleus can be identified by constructing phylogenetic trees containing both eukaryotic and prokaryotic homologs and then looking for trees in which *Arabidopsis* and cyanobacteria branch together. The average protein length for three selected cyanobacteria (*Nostoc* sp. PCC7107, *Prochlorococcus marinus*, and *Synechocystis* sp. PCC 6803) was 314 aa, whereas the average length for *Arabidopsis* nuclear proteins was 406 aa. Similar to previous studies [8], [34], [36], we identified 1339 putative *Arabidopsis* nuclear proteins of cyanobacterial origin. Those endosymbiotic proteins had an average size of 473 aa, which was significantly larger than the average size of their cyanobacterial orthologs (314 aa; Paired Wilcox test, *P* < 0.001) and also larger than the average size of all *Arabidopsis* proteins (406 aa). Therefore, these results clearly exclude the “bacterial gene migration” hypothesis as the explanatory cause for the average smaller size of plant proteins. On the contrary, gene migration from the chloroplast genome to the nuclear genome led to a slight increase of protein size. On average, endosymbiotic plant proteins were ∼159 aa larger than the cyanobacterial orthologs, possibly arising from the need for additional regulatory domains [5], [37]. Therefore, the endosymbiotic origin of plastids cannot account for the shorter average length of proteins within all Streptophyta species.

### The average size of proteins varies according to cellular compartmentation

The third possible explanation as to why plant proteins are smaller than Metazoa or Fungi could be their localization in different cellular organelles. Compared to animal and fungal cells, plants have additional subcellular compartments, *e.g.*, chloroplast, vacuole, and specialized peroxisomes. It is not known whether cell subcompartmentalization significantly affects the size distribution of proteins in eukaryotic species. In order to answer this question, we calculated the median and average size of proteins localized in different subcellular compartments. For this purpose, we analyzed the *Arabidopsis* genome using the Gene Ontology (GO) Slim classification of cellular component. It must be noted at this point that the proteolytic shortening of proteins, *e.g.*, due to subcellular import into chloroplasts, was not accounted for in this study since our aim was not to analyze the final sizes of the processed polypeptides, but rather the protein lengths as defined by the nuclear DNA-encoded proteome of each subcellular compartment. Therefore, the requirement for transit peptides (size ∼10–50 aa) should increase protein size (difference of digital proteome compared to the physical processed end proteins).

The median and average size of proteins grouped by GO categories was significantly (*P* = 2.2E–16) different for various compartments in *Arabidopsis* (Table 3). The largest proteins were the membrane proteins (plasma membrane, Golgi apparatus, and other membranes GO groups), whereas the smallest proteins corresponded to the GO groups of ribosome, unknown cellular component, endoplasmic reticulum (ER) and extracellular proteins (Table 3). There were no significant size differences among cytosolic, plastidial, nuclear and mitochondrial proteins (Table 3). These data suggest that the presence of transit peptides in chloroplast genes (∼10–50 aa) did not significantly increase average protein size compared to cytosolic proteins (without targeting signals). Plastidial proteins were neither particularly large nor small compared to other proteins. Similar results were obtained for mean and median protein sizes in rice according to GO grouping (data not shown).

Overall, these results do not support the hypothesis that the smaller average size of plant proteins is caused by prevalence of plastidial proteins in comparison to animal cells that lack chloroplasts. Plants might have fewer membrane proteins (of large size;>500 aa) but instead have more ribosomal, vacuolar, extracellular, and unknown proteins (of small size; < 250 aa) compared to fungi and animals. In order to clarify this possibility, we analyzed protein compartmental distribution in other model organisms after grouping into GO categories. Since GO slim categories were not available for animal genomes, we used the Map2Slim script to map *Homosapiens* GO to GO slim generic annotation. We first compared a model plant (*Arabidopsis*, Table 3) to the human genome (Table 4). Our data showed that protein lengths were mostly smaller in plants than in humans. Comparison of protein size medians from different cellular components in *Arabidopsis* and humans, such as the extracellular matrix (400 aa *vs.* 755 aa), Golgi (431 aa *vs.* 495 aa), cytoplasm (348 aa *vs.* 491 aa), cytosol (367 aa *vs.* 451 aa), nucleus (325 aa *vs.* 472 aa), indicated larger proteins in Metazoa. Proteins from mitochondria (341 aa *vs.* 312 aa) and ribosome (206 aa *vs.* 180 aa) were roughly similar in both species. Proteins from 6 out of 11 GO cellular component categories were larger in humans than those in *Arabidopsis*, whereas no GO category contained significantly larger proteins in plants.

We also compared proteins from *Arabidopsis* (Table 3) with those from a model fungus, Baker’s yeast (Table 5). Membrane proteins are larger in comparison to ribosomal proteins (Table 3, Table 4, Table 5). Membrane proteins accounted for 16.4% of total proteins in *Arabidopsis* and 20.5% in yeast, whereas ribosomal proteins accounted for 1.1% of total proteins in *Arabidopsis* and 2.5% in yeast. This revealed that the number of proteins (as percentage basis) falling within a GO cellular component category had a rather modest impact on average protein size. Plants have not a higher percentage of small ribosomal proteins compared to yeast (Table 3, Table 5). Instead, proteins belonging to a specific GO cellular component category were mostly larger in the fungal (Table 5) than in the plant species (Table 3; Figure S2). In total, 9 out of 11 GO cellular component categories contained larger proteins in yeast, while the remaining 2 categories (unknown and ribosome) behaved similarly in both species and no GO category contained larger proteins in the plant.

Overall, the data indicate that across different subcellular compartments, proteins from plants are in general smaller than human and yeast proteins (Table 3, Table 4, Table 5; Figure S2).

### Protein length is related to exon structure

**Figure 3:**
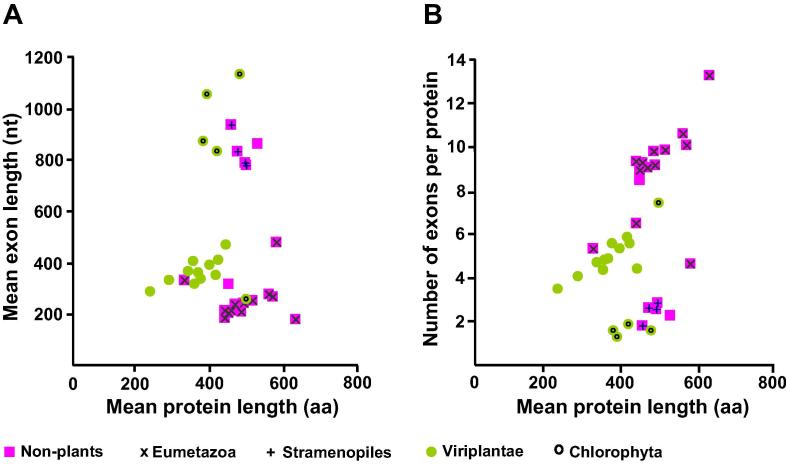
**Relation between exon size, exon number and protein length in species of dataset 1A.** Average exon length. **B.** Average exon number per protein. The genomic information from representative plant and animal genomes was selected according to data availability as described previously [7]. Average exon length (of all exons from a particular species) and mean exon number per protein was calculated for each species and plotted against the mean protein size for the same species. All plants are indicated in green, while all non-plants are indicated in purple (regardless of the inner symbol). Unicellular plants (Chlorophyta) and other unicellular species (Stramenopiles) are indicated with inner symbols ο and +, respectively. Multicellular animals (Eumetazoa) are indicated with inner symbol ×.

**Figure 4:**
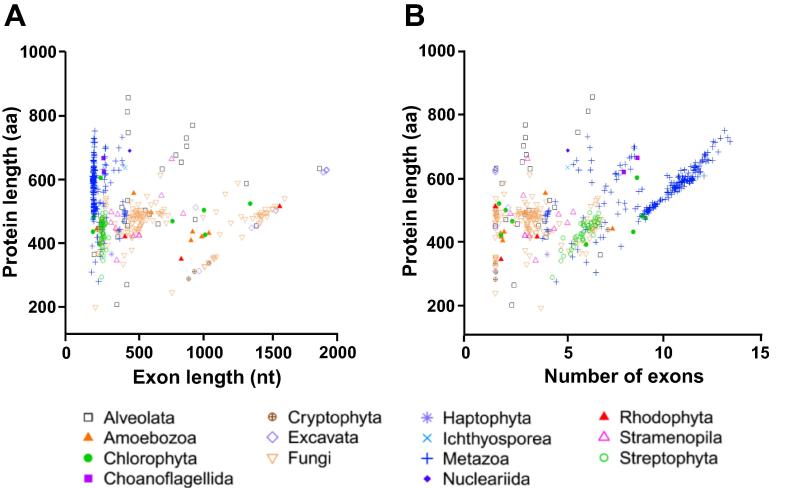
**Relation between protein length and exon structure in dataset 3** Scatter-plot for 492 eukaryotic species divided in 14 phylogenetic groups. The genomes were obtained from the RefSeq release 70 (dataset 3). **A.** Average exon numbers were plotted against average protein length in 14 phylogenetic groups. **B.** Average exon length was plotted against average protein length in 14 phylogenetic groups. Each dot represents the average of one particular species. Phylogenetic groups are indicated with different symbols as shown in the figure.

The fourth possible explanation is the nature of eukaryotic genes being divided into introns and exons, whereas exons can sometimes correspond to specific protein domains. It can be intuitively postulated that protein length would be strongly affected by the exon features. We therefore analyzed average exon length and average exon number per gene in all the eukaryotic genomes of datasets 1 and 3. For dataset 1, average exon length in nucleotides (nt) was obtained by averaging length of all exons from each gene first, and then calculating a global average across all genes of each species and then the mean exon length was plotted against the mean protein length (Figure 3). For dataset 3, average exon length was calculated for 492 species without prior averaging (Figure 4). Table 2 shows summary statistics of protein length, exon number, and exon length in each of the 14 phylogenetic groups of dataset 3.

Most animal species had proteins with ∼10 exons of average size ∼210 nt (Figure 3, Figure 4). Plants had genes with fewer exons (4–6 per protein) but the mean exon length was larger (∼380 nt) (Figure 3). All animal and plant species are multicellular organisms, whereas results varied according to cellularity. Unicellular species (*e.g.*, Chlorophyta and Stramenopiles) had proteins encoded by an average of only ∼2 exons, however, their exons were much larger on average (∼900 nt per exon) (Figure 3, Figure 4). In contrast to the small exon size of animal (176 nt) and the medium exon size of plants (230 nt) (Table 2), a high number of fungal species had large exons (∼1300 nt) (Figure 4). Many unicellular eukaryotes (*e.g.*, Excavata, Nucleariida, Alveolata, Amoebozoa, and Rhodophyta) also had larger exons than plant species (Table 2 and Figure 4). Therefore, average protein size across all species of dataset 3 was neither significantly correlated to average exon number only nor to exon length only.

### There is a non-linear relationship between exon number and exon size

**Figure 5:**
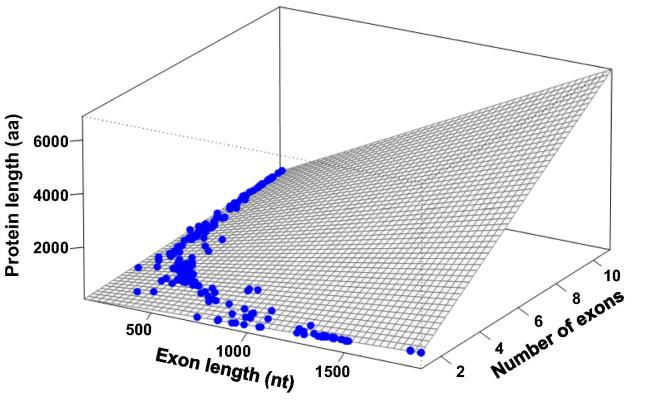
**Linear regression model of protein length in dataset 3** The averages of protein size, exon length (EL), and exon number (EN) from each species in dataset 3 were projected into a 3-dimensional space plotting EL on the *X*-axis, EN on the *Z*-axis, and protein size on the *Y*-axis. A linear regression model was constructed to fit the data. The formula of the optimally-fitted hyperplane is *Y* = −1.03 + 0.000034 EL + 0.34 EN + 0.33 EL × EN. *Y* indicates protein length. The hyperplane formula allows predicting protein length using data from Table 2 (492 species in total). Correlation value *R*^2^ ≈ 1.

We then tested whether the final protein length would result from a factorial multiplication of both exon number and length. A curved hyperplane shows how exon length and number both contribute to protein length (Figure 5). The non-linear relationship between exon length, exon number and protein size can be plotted in a characteristic curved hyperplane (Figure 5).

Plants have exons larger than animals but smaller than fungi (Figure 4, Table 2). The largest exons (1330 nt) were found for the Excavata group, whereas the smallest exons for the Metazoa group (176 nt) (Table 2). The biggest proteins were found for the Nucleariida group (690 aa) whereas Haptophyta group (367 aa) had the smallest proteins (Table 2, Figure 2). Different phylogenetic lineages appear to utilize one or the other strategy to attain large protein: large exons (*e.g.*, Excavata and Rhodophyta) or numerous exons (*e.g.*, Metazoa and Choanoflagellida) (Table 2; Figure 4). Multicellular plants rather utilize an intermediate strategy, whereas fungal species display a much wider range of strategies to attain larger proteins, both with medium (∼500 nt) and large (∼1500 nt) exons (Figure 4A).

### A linear model explains the relationship between protein length and the exon features

A linear regression model was applied to dataset 3 considering the number and length of exons as predictors of protein length (Figure 5). Both exon length (EL) and exon number (EN) were significant (*P* < 0.001; *R*^2^ = 0.5) in the model. When the interaction terms (EN × EL) were included in the model, the *R*^2^ value was ∼1 which is extremely high (Figure 5). Analysis of dataset 1 yielded similar results for exon number and length (Figure 3), whereas dataset 2 lacked information on exon features.

As Felstenstein and others have pointed out, an observed correlation may be spurious if phylogenetic relationships are ignored [38], [39], [40]. As a first step to correct this bias and to perform a phylogenetical independent contrasts (PIC) analysis, we searched for small ribosomal RNA (srRNA) sequences in two curated databases named SINA and PRR2 [41], [42]. We found 233 srRNA sequences that were present also in our dataset 3 at least at ‘genus’ level (see Methods). After phylogeny reconstruction by ML model, we removed 12 species from the tree because their branch lengths were zero, which is not useful for PIC analysis. Moreover, a rooted tree is mandatory to accomplish PIC analysis. Since the real root for the eukaryotes is not known yet, we used the ‘midpoint root’ criteria to put a root to our obtained phylogeny. This resulted in a root between Excavata and the others. Then, PIC analysis was conducted and the resulting contrast data were used to adjust a linear model. In this case, the *R*^2^ obtained was only 0.67. The number of exons was highly significant (*P* = 2E–6), whereas exon length (*P* = 0.12) and the interaction term (*P* = 0.19) were not significant. This clearly shows that during the course of evolution, the number of exons has been much more important to determine protein length than the size of the individual exons.

### An average plant protein has fewer domains than an average animal protein

**Figure 6:**
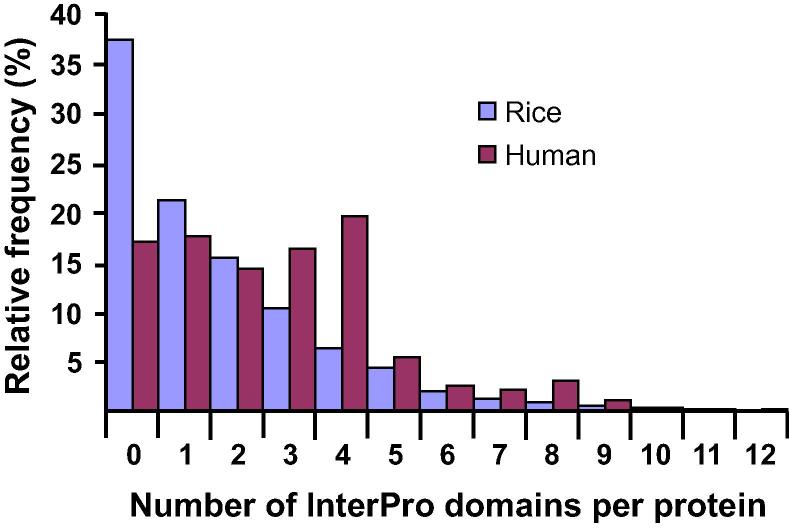
**Number of InterPro domains per protein in an independent Pfam dataset** Multidomain proteins are found more frequently in animals than in plants. The number of domains per proteins was calculated (for each gene individually) from a model plant (*O. sativa*) and a model animal species (*H. sapiens*). Original data were downloaded from the Pfam and InterPro databases on 10 March, 2016. The data on the number of domains per gene were obtained by parsing and processing the files with in-house developed Perl and R scripts. The histogram was constructed in Excel using the relative frequency. *Y*-axis indicates the percentage of proteins containing a certain number of domains among the total proteins (*X*-axis).

Results from all 3 datasets suggest that Metazoa and Fungi have both larger proteins than Streptophyta. In comparison to animal species (10.1 exons per gene), plants have 44% fewer exons per gene but 31% larger exons (Table 2). As a result, plant proteins are 27% smaller on average than animal proteins (Table 2 and Figure 2). We speculate that this may be indicative of plant proteins consisting of fewer functional domains than animal proteins. In order to test it, we counted the number of Pfam and InterPro domains per protein in 2 representative models: the human and rice genomes. Up to 35% of the human proteins contained ⩾ 4 domains, whereas only 16% of the rice proteins had ⩾ 4 domains (Figure 6). Moreover, up to 5.1% of the human proteins contained ⩾ 8 domains, whereas only 2.0% of the rice proteins had ⩾ 8 domains (Figure 6). Comparison of domain distribution histograms revealed that animals not only have larger proteins but also contain more functional domains than plants.

## Conclusions

Larger proteins can accommodate more functional and regulatory domains than smaller proteins. Plants have a higher number of coding genes than other species (Table 1), but animal proteins are larger and presumably also more complex (multidomain proteins). Plant proteins are ∼22% larger on average than bacterial proteins but they are also ∼27% smaller on average than animal proteins. We confirmed that proteins unique to plants are shorter than plant proteins conserved among other eukaryotic lineages. Through an exhaustive statistical analysis, we explored several possible explanations and ruled out that the smaller size of plant proteins was caused by cyanobacterial endosymbiosis. We demonstrated that average protein length varies according to subcellular compartment. Size differences were noted as a systematic shift across all protein lengths (Figure S1) and across several cellular compartments in plants, animals, and fungi (Table 3, Table 4, Table 5; Figure S2).

**Figure 7:**
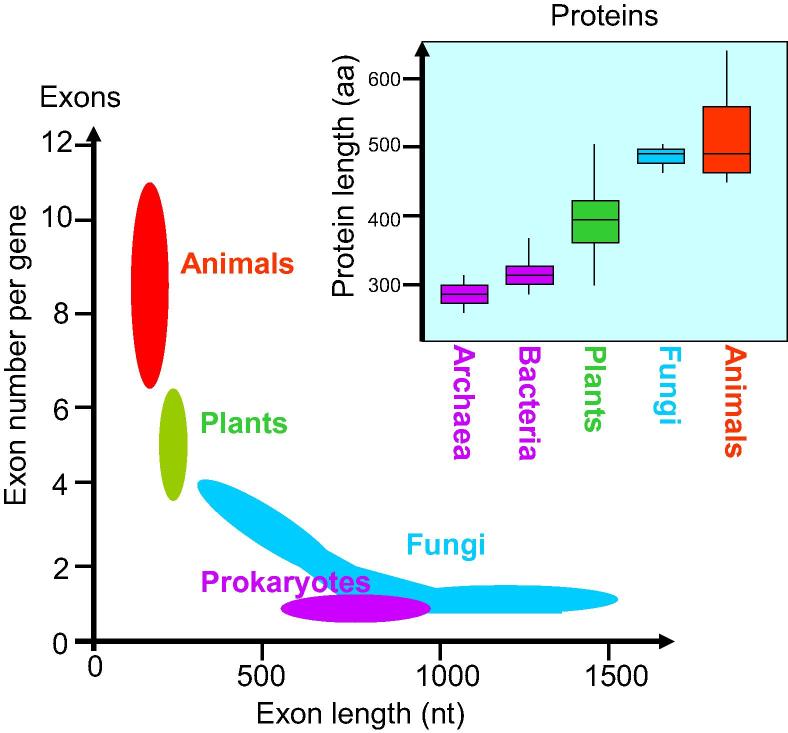
**Comparison of proteome features between different organisms** Using the combined results from datasets 1–3, a simplified model was manually constructed for different phylogenetic groups including Archaea, Bacteria, Streptophyta, Metazoa, and Fungi. The figure visualizes the preferred strategy of eukaryotes for attaining larger proteins than green plants: either using more exons (animals, red) or larger exons (fungi, blue). Prokaryotes (purple) use the “one exon strategy” and therefore have smaller proteins than eukaryotes, and plants (green) stand somehow in the middle.

On the one hand, plant proteins are smaller because they are encoded by fewer exons and contain fewer domains than animal proteins. On the other hand, fungal proteins are larger than plant proteins because fungal exons are much larger on average than those of plants (Figure 7). It is open for debate whether fungal proteins are more or less complex than plant or animal proteins [29]. The fact is that fungal genomes code for fewer proteins than animal or plant genomes (Table 1). We found it remarkable that myceliar or unicellular species (*e.g.*, Fungi, Excavata, Alveolata, and Rhodophyta) with low cellular differentiation have few but rather large exons (Table 2), whereas multicellular organisms with different types of cells, tissues, and organs (Metazoa and Streptophyta) employ many but rather small exons to code for their genes (Figure 4). It seems that unicellular undifferentiated eukaryotes (*e.g.*, Fungi, Excavata, Rhodophyta, and Stramenopila) are similar to prokaryotes (Archaea and Bacteria) that lack introns and thus have large exons (only 1 “exon” per gene) (Figure 7).

In summary, our modeling results expand previous studies that have suggested a positive relationship between the number of exons and protein length within a limited number of species [43]. It also confirms the positive association between average protein size and average number of domains [4]. Our linear regression analysis also shows that for each additional protein domain, a sequence length of ∼39 aa is added on average to a eukaryotic protein of size ∼367 aa. Therefore, the small size difference between a protein of ∼436 aa (*e.g.*, photosynthetic plant) and a protein of ∼595 aa (*e.g.*, heterotrophic animal) may determine an increase of regulatory complexity of having only 1 domain or 5 domains. Thus, the shorter length of plant proteins is not a trivial mathematical fact but may have profound biological implications. It seems that the average number of exons (and not exon length) is correlated somehow with the capacity of cellular differentiation of the organism. It is highly interesting what can be revealed by statistical analysis of the digital proteomes of the different phylogenetic lineages and we therefore recommend further investigations of plant genomes at a greater depth combining the efforts from several groups in the proteomics and evolutionary fields.

## Methods

### Construction and curation of datasets

Protein sequences of selected organisms from datasets 1 and 2 were obtained and curated as described previously [7]. In addition, a third dataset of eukaryotic species (dataset 3) was constructed using the GenBank files downloaded during July 2015 from the NCBI RefSeq release 70 [44].

After downloading and parsing the GenBank files, we obtained a dataset with ∼9.6 million sequences represented by 5837 species. However, only a small number of sequences are reported for many species. To avoid bias in our statistical analysis due to small sample size, we only retained species having at least 500 sequences. This threshold was considered mathematically reliable since the lognormal distribution of protein sizes allows estimating minimal sampling sizes (N ⩾ 500) to attain minimal confidence levels (⩽4.5%) for estimating the true means and medians [7]. The final dataset 3 had ∼9.5 million proteins and ∼74.4 million exon sequences from 492 species divided in 14 phylogenetic groups (Table 1).

The exon features of dataset 3 were extracted from the coding determining sequence (CDS) lines in GenBank files as described elsewhere [43]. For example, the CDS of XP_007325329.1 in *Agaricus bisporus* join (18,372 to 18,786, 18,829 to 19,191, and 19,447 to 19,622) consists of three exons with lengths of 415 bp, 363 bp, and 176 bp, respectively. Using a stringent quality control, we excluded all CDSs with ambiguous exon boundaries, start or stop codons. For instance, proteins that do not start explicitly with methionine (∼1%) were excluded. As a result, a small percentage of CDSs was excluded in different groups (3.6% in Protist, 1.44% in Fungi, 0.96% in Streptophyta, and 0.87% in Metazoa).

### Statistical analysis

Median values and arithmetic averages of protein length, exon length, and number of exons were calculated for each of the 492 species in dataset 3. To obtain the mean number of exons per gene, we first counted the total number of exons and then divided it by the number of protein sequences considered. Mean exon length was obtained in a similar fashion by first summing the length of all exons and then dividing by the total number of exons.

To compare protein length between species or phylogenetic groups, we first applied the Kruskal–Wallis test [45] at *P* < 0.05 and then performed pairwise comparisons with a local implementation of Kruskal–Wallis post-test [46]. The non-parametric Kruskal–Wallis test [45] is more robust than a Tukey test, since it considers all the individual values of the distribution of sizes and not just a single value for mean or median (Table 2, Table 3, Table 4, Table 5). *P* values were adjusted with the false discovery rate (FDR) method [47] and labels of multiple comparisons were assigned using the “multcompView” package [48]. All statistical analyses were performed using R software [49].

### Phylogenetic regression analysis

The evolutionary analysis was performed based on the established and best curated taxonomy of eukaryotes [19], [20], [21]. The results were validated by performing an independent reconstruction of the phylogenies using the sequences of the small subunit rRNAs of 233 representative eukaryotic species. We consulted the Protist Ribosomal Reference database (PR2) [41] and the SILVA database [42] (http://ssu-rrna.org) for phylogenetic regression analysis. Small subunit rRNA sequences were aligned with SINA [50]. Gaps of multiple sequence alignments were eliminated using trimAl [51] with the “automated1” option. Both estimation of the best-fit model and reconstruction of the phylogenetic tree were inferred with jModelTest version 2.1.7 [52], using the maximum likelihood model through PhyML [53]. The resulting tree was rooted using the “phangorn” R package [54].

Phylogenetic independent contrast regression analysis [40] was conducted using the “ape” R package [55]. Linear model was forced through the origin and adjusted as recommended by Garland et al [56]. Response variable was protein length and explanatory variables were exon number and exon length.

### Identification of cyanobacterial orthologs of nuclear proteins in *Arabidopsis*

Sequences of nuclear-encoded proteins from the whole genomes of 4 archaebacteria (*Pyrococcus furiosus*, *Methanobacterium* AL, *Methanococcus maripaludis*, and *Archaeoglobus fulgidus*), 3 Gram positives (*Mycoplasma genitalium*, *Bacillus subtilis*, and *Mycobacterium* sp. JDM601), 3 cyanobacteria (*Nostoc* sp. PCC7107, *P.marinus*, and *Synechocystis* sp. PCC6803), 4 eubacteria (*Borrelia afzelii*, *Treponema azotonutricium*, *Chlamydia pecorum*, and *Aquifex aeolicus*), and 4 proteobacteria (*Rickettsia akari*, *Helicobacter acinonychis*, *Haemophilus ducreyi*, and *Escherichia coli*) were obtained from NCBI genome database in August, 2015. *A. thaliana* and *Saccharomyces cerevisiae* nuclear proteomes were downloaded from The *Arabidopsis* Information Resource (TAIR) (https://www.arabidopsis.org) and Saccharomyces Genome Database (SGD) (http://www.yeastgenome.org), respectively.

A non-redundant set of *Arabidopsis* sequences was obtained with the Cluster Database at High Identity with Tolerance (CD-HIT) program using default parameters [57], [58]. Construction of BLAST tables was done with the reciprocal best BLAST hits by comparing *Arabidopsis* proteome with all other proteomes with thresholds of *e*-value <10^−10^ and aa sequence identities >30%. Multiple sequence alignments (MSAs) of proteins were obtained with the multiple sequence comparison by log-expectation (MUSCLE) [59] using default parameters. Gaps were removed using trimAl [51] with the “gappyout” option. Phylogenetic trees were reconstructed with PhyML using a maximum likelihood approach [60]. The best-fit model was inferred with ProtTest [61]. All the procedures above were conducted using the Environment for Tree Exploration (ETE) pipeline for phylogenetic analysis [62]. To identify genes of endosymbiotic origin that migrated from the chloroplast to the nucleus in *Arabidopsis*, we searched for phylogenetic trees in which cyanobacterial protein sequences branch together with *Arabidopsis* nuclear protein sequences [8], [34].

### Analysis of protein length between GO categories of yeast and *Arabidopsis*

The GO Slim annotations were downloaded from TAIR and SGD. *H. sapiens* and *O. sativa* GO annotations were obtained from the Gene Ontology Consortium database (http://geneontology.org) and then mapped to GO Slim generic annotations using the Map2Slim program, which is available from the Comprehensive Perl Archive Network (CPAN) through the go-perl package. Statistical comparisons between organisms/compartments were performed by non-parametric Kruskal–Wallis [45] test at *P* < 0.05 and post hoc analysis or by analysis of variance (ANOVA) and Tukey’s post hoc tests.

## Authors’ contributions

AT conceived the study, coordinated the project, participated in the statistical analysis, prepared most figures, contributed to the biological interpretation of the results, and wrote the manuscript. ORS and PPR performed statistical analysis, wrote Perl and R scripts, and prepared some figures and tables. LDA wrote some perl scripts for sequence analysis and contributed to the evolutionary interpretation of the data. All authors revised and approved the final manuscript.

## Competing interests

The authors declare no competing interests.

## Figures and Tables

**Table 1:** Phylogenetic coverage and global features of proteome dataset 3

**Group**	**No. (%) of species**	**Total No. of proteins**	**Average No. of proteins/species**	**Total No. of exons**	**Average No. of exons/species**
Alveolata	24 (4.9)	166,806	6950	596,916	24,872
Amoebozoa	7 (1.4)	70,337	10,048	218,096	31,157
Chlorophyta	9 (1.8)	78,172	8686	424,697	47,189
Choanoflagellida	2 (0.4)	19,817	9909	164,858	82,429
Cryptophyta	4 (0.8)	22,383	5596	146,349	36,587
Excavata	12 (2.4)	160,927	13,411	171,251	14,271
Fungi	143 (29.1)	1,303,212	9113	4,285,632	29,969
Haptophyta	1 (0.2)	31,735	31,735	118,186	118,186
Ichthyosporea	1 (0.2)	8510	8510	40,799	40,799
Metazoa	228 (46.3)	5,743,160	25,189	58,003,499	254,401
Nucleariida	1 (0.2)	6115	6115	29,416	29,416
Rhodophyta	3 (0.6)	21,255	7085	39,453	13,151
Stramenopila	11 (2.2)	175,058	15,914	614,663	55,878
Streptophyta	46 (9.3)	1,692,582	36,795	9,571,726	208,081
Total	492 (100)	9,522,269	19,354	74,425,541	151,271

*Note*: Proteome dataset 3 was constructed from the RefSeq database release 70 representing 492 species. Phylogenetic groups are ordered alphabetically. Phylogenetic clades included are “Opisthokonta a” (Ichthyosporea, Choanoflagellida, and Metazoa), “Opisthokonta b” (Nucleariida and Fungi), “SA” (Stramenopiles and Alveolata), “Other protists” (Excavata, Cryptophyta, Haptophyta, and Amoebozoa), and “Archaeplastida” (Rhodophyta, Chlorophyta, and Streptophyta).

**Table 2:** Protein size, exon size, and exon number in proteome dataset 3

**Group**	**Protein length (aa)**			**Exon number**			**Exon length (nt)**		
	**Mean**	**Median**	**K**	**Mean**	**Median**	**K**	**Mean**	**Median**	**K**
Alveolata	535	364	e	3.6	2	i	449	161	h
Amoebozoa	463	351	f	3.1	2	j	448	192	f
Chlorophyta	490	369	e	5.4	3	g	270	139	j
Choanoflagellida	648	459	b	8.2	6	b	237	113	m
Cryptophyta	435	316	h	6.5	5	c	199	82	n
Excavata	471	334	g	1.1	1	m	1330	935	a
Fungi	462	381	d	3.3	2	k	421	195	e
Haptophyta	367	294	i	3.7	3	h	295	164	g
Ichthyosporea	637	494	a	4.8	4	d	398	152	i
Metazoa	595	439	c	10.1	7	a	176	126	l
Nucleariida	690	499	a	4.8	4	e	429	216	c
Rhodophyta	411	334	g	1.9	1	n	665	330	b
Stramenopila	467	356	f	3.5	2	l	399	196	d
Streptophyta	436	363	f	5.7	4	f	230	128	k

*Note*: Significant differences in protein size, exon size, and exon numbers between different phylogenetic groups were calculated based on the Kruskal–Wallis test (*P* < 0.05) and are indicated using different letters in the “K” columns. Phylogenetic groups are ordered alphabetically.

**Table 3:** Size of *Arabidopsis* proteins in different cell components

**Category**	**No. of proteins**	**Protein length (aa)**			
		**Median**	**Mean**	**SD**	**K**
Plasma membrane	2152	446	518	336	a
Golgi apparatus	126	431	510	277	a, b
Other membranes	3084	390	453	318	b, c
Cytosol	1700	367	444	333	c, d
Cell wall	549	400	436	220	c, d
Chloroplast	3446	359	431	311	c, d, e
Plastid	1228	361	419	294	c, d, e, f
Other intracellular components	3969	354	425	317	c, d, e, f
Other cytoplasmic components	3063	348	411	296	c, d, e, f
Nucleus	1810	325	416	305	c, d, e, f
Mitochondria	770	341	389	302	d, e, f
Other cellular components	4612	322	376	340	e, f
Extracellular	477	316	375	269	e, f
Endoplasmic reticulum	213	321	372	226	f
Unknown cellular components	4468	237	298	236	g
Ribosome	356	206	248	169	g

*Note:* Proteins were grouped by Gene Ontology category “cellular component” according to GO Slim terms. Significant differences in protein size between different phylogenetic groups were calculated based on Kruskal–Wallis test (*P* < 0.05) and are indicated using different letters in “K” column. Categories are ordered according to median protein size. SD, standard deviation.

**Table 4:** Size of human proteins in different cell components

**Category**	**No. of proteins**	**Protein length (aa)**		
		**Median**	**Mean**	**K**
Proteinaceous extracellular matrix	461	755	1146	a
Microtubule organizing center	993	657	916	b
Nuclear envelope	1028	656	916	b
Chromosome	896	602	787	c, d
Cilium	1216	602	883	c, d
Cytoskeleton	2405	597	891	c
Cell	4176	565	752	d
Golgi apparatus	3291	495	975	e
Cytoplasm	8623	491	720	f
Endoplasmic reticulum	3291	484	624	g, h, i
Protein complex	6431	480	683	g
Intracellular	2981	479	635	g, h
Nucleolus	1033	473	621	g, h, i, j, k
Nucleus	7649	472	617	h, i
Nucleoplasm	14,012	466	640	j
Cellular component	8857	463	658	g, h
Nuclear chromosome	735	462	686	e, f, g, h
Plasma membrane	16,591	457	610	i, j
Endosome	1768	456	606	g, h, i, j
Cytosol	17,841	451	629	k
Vacuole	165	446	586	i, j, k, l, m
Lipid particle	70	441	546	e, f, g, h, i, j, k, l, m
Organelle	6957	427	657	l
Peroxisome	345	424	525	f, g, h, i, j, k, l
Cytoplasmic, membrane bounded vesicle	2121	418	599	l
Lysosome	995	390	539	m
Extracellular space	1949	355	512	n
Mitochondrion	4291	312	382	o
Extracellular region	15,696	216	436	p
Ribosome	346	180	232	q

*Note:* Human proteins were grouped by Gene Ontology category “cellular component” using Map2GO. Significant differences in protein size between different phylogenetic groups were calculated based on Kruskal–Wallis test (*P* < 0.05) and are indicated using different letters in “K” column. Categories are ordered according to median protein size.

**Table 5:** Size of yeast proteins in different cell components

**Category**	**No. of proteins**	**Protein length (aa)**			
		**Median**	**Mean**	**SD**	**K**
Site of polarized growth	247	666	741	436	a
Cellular bud	207	656	730	442	a, b
Cell cortex	146	637	765	536	a, b
Plasma membrane	390	568	632	383	b, c
Microtubule organizing center	71	494	635	534	b, c, d, e
Cytoskeleton	204	558	655	474	c, d
Vacuole	274	497	570	376	d, e
Chromosome	374	482	587	415	d, e
Golgi apparatus	196	468	570	403	e, f
Other	73	433	488	285	e, f, g
Peroxisome	71	394	470	242	e, f, g
Extracellular region	28	388	486	260	e, f, g
Membrane	1611	453	538	390	f
Endomembrane system	790	437	533	396	f
Nucleus	1988	426	524	386	f
Nucleolus	251	428	517	400	f, g
Cell wall	96	414	482	325	f, g
Cytoplasm	3707	405	499	376	g
Endoplasmic reticulum	409	403	477	359	g
Mitochondrion	1110	390	507	450	g
Mitochondrial envelope	354	307	340	207	h
Unknown cellular component	679	222	297	278	i
Ribosome	340	212	335	335	i

*Note:* Yeast proteins were grouped by Gene Ontology category “cellular component” with GO Slim terms. Significant differences in protein size between different phylogenetic groups were calculated based on Kruskal–Wallis test (*P* < 0.05) and are indicated using different letters in “K” column. The K test is statistically more robust than a Tukey test (see Table 3). Categories are ordered according to median protein size. SD, standard deviation.

## Supplementary material

Supplementary Figure S1 Density curve of protein length distribution of the proteomes in dataset 3 All animal proteins (blue solid line, N = 5,743,160) and all plant proteins (green dotted line, N = 1,692,582) were pooled regardless of the species. Protein length was transformed logarithmically. The X-axis indicates protein length (aa) scaled in natural logarithmus (Ln), whereas the Y-axis indicates the density (estimated probability) that a randomly selected protein from animals or plants has that given length.

Supplementary Figure S2 Comparison of protein size between *Arabidopsis* and Baker’s yeast Protein size was calculated from all proteins belonging to a particular GO category of cellular component. The diagonal line shows exact correspondence across categories. Points above the line indicate that yeast proteins were larger than *Arabidopsis* proteins. Points below the line (shaded triangle) would indicate that yeast proteins are smaller, which was not the case for any GO category. **A**. Median protein size. **B**. Average protein size. Number of proteins ranges 126–2152 for *Arabidopsis* and 28–3707 for yeast, respectively.
