# Supplementary figures and images for: Plant Proteins Are Smaller Because They Are Encoded by Fewer Exons than Animal Proteins
Source: Genomics Proteomics Bioinformatics. 2016 Dec 18;14(6):357–70. doi: 10.1016/j.gpb.2016.06.003 (PMC5200936; doi:10.1016/j.gpb.2016.06.003)

## Slide 1
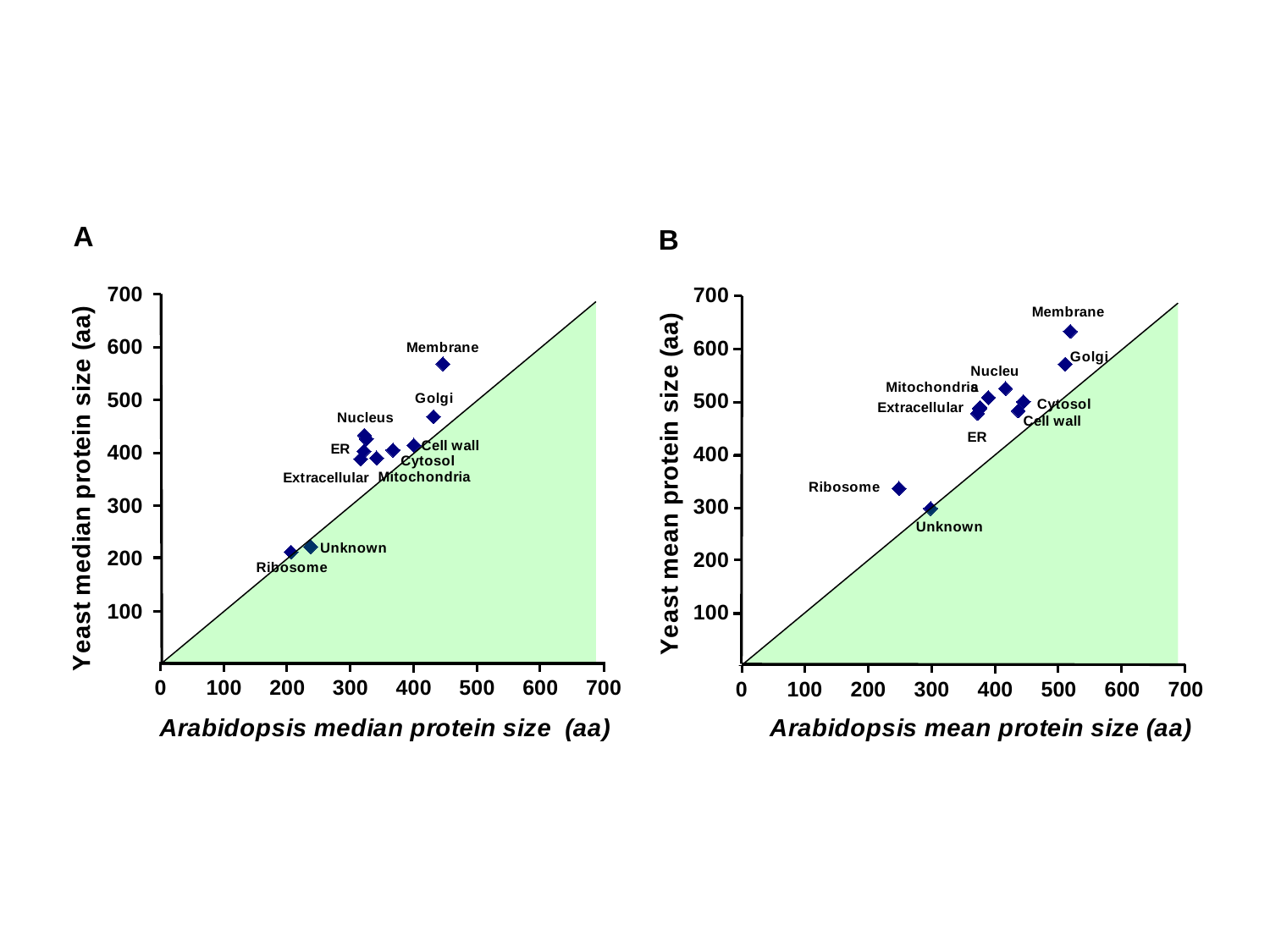

### Chart
| Category | |
|---|---|
### Chart
| Category | |
|---|---|

Supplement: Supplementary Figure S1 — Density curve of protein length distribution of the proteomes in dataset 3 All animal proteins (blue solid line, N = 5,743,160) and all plant proteins (green dotted line, N = 1,692,582) were pooled regardless of the species. Protein length was transformed logarithmically. The X-axis indicates protein length (aa) scaled in natural logarithmus (Ln), whereas the Y-axis indicates the density (estimated probability) that a randomly selected protein from animals or plants has that given length. [file mmc1.pptx]

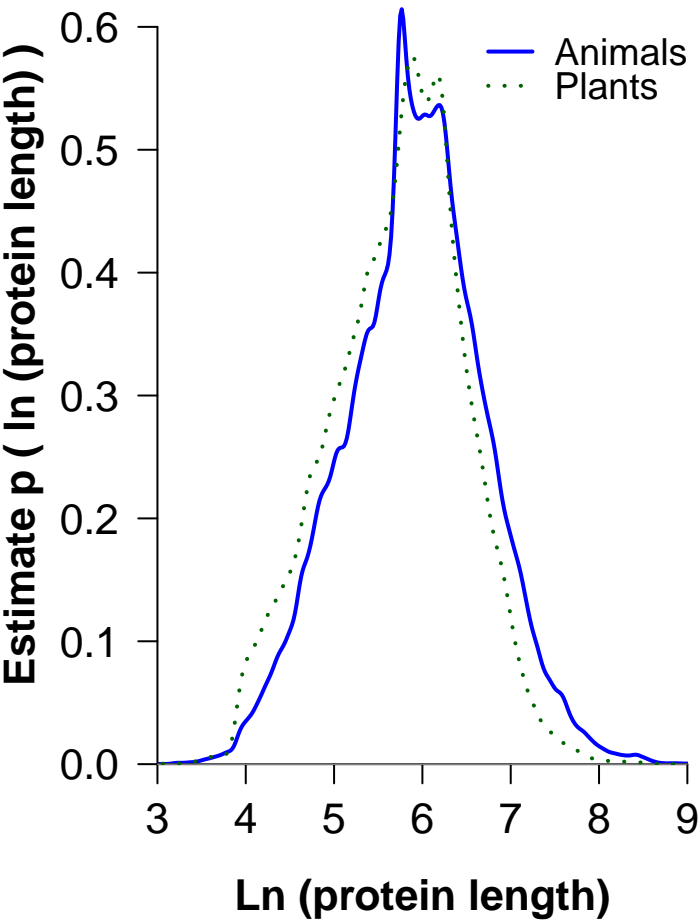

Supplement: Supplementary Figure S2 — Comparison of protein size between Arabidopsis and Baker’s yeast Protein size was calculated from all proteins belonging to a particular GO category of cellular component. The diagonal line shows exact correspondence across categories. Points above the line indicate that yeast proteins were larger than Arabidopsis proteins. Points below the line (shaded triangle) would indicate that yeast proteins are smaller, which was not the case for any GO category. A. Median protein size. B. Average protein size. Number of proteins ranges 126–2152 for Arabidopsis and 28–3707 for yeast, respectively. [file mmc2.pdf]
